# A phenotypic screening approach to target p60AmotL2-expressing invasive cancer cells

**DOI:** 10.1186/s13046-024-03031-w

**Published:** 2024-04-09

**Authors:** Pedro Fonseca, Weiyingqi Cui, Nona Struyf, Le Tong, Ayushi Chaurasiya, Felipe Casagrande, Honglei Zhao, Dinura Fernando, Xinsong Chen, Nicholas P. Tobin, Brinton Seashore-Ludlow, Andreas Lundqvist, Johan Hartman, Anita Göndör, Päivi Östling, Lars Holmgren

**Affiliations:** 1 Department of Oncology and Pathology, Karolinska Institutet, U2, Bioclinicum J6:20, Solnavägen 30, 171 64 Solna, Stockholm Sweden; 2 Science for Life Laboratory, Tomtebodavägen 23a, 171 65 Stockholm, Sweden; 3 Breast Center, Karolinska Comprehensive Cancer Center, Karolinska University Hospital, Stockholm, Sweden; 4 Department of Clinical Molecular Biology, University of Oslo, Akershus Universitetssykehus, 1478 Lørenskog, Oslo, Norway

**Keywords:** Cancer, Cancer invasion, Metastasis, Mechanotransduction, Phenotypic drug screening, Patient-derived organoids, AmotL2, p60AmotL2, BET inhibitors, Nascent RNA-seq

## Abstract

**Background:**

Tumor cells have the ability to invade and form small clusters that protrude into adjacent tissues, a phenomenon that is frequently observed at the periphery of a tumor as it expands into healthy tissues. The presence of these clusters is linked to poor prognosis and has proven challenging to treat using conventional therapies. We previously reported that p60AmotL2 expression is localized to invasive colon and breast cancer cells. In vitro, p60AmotL2 promotes epithelial cell invasion by negatively impacting E-cadherin/AmotL2-related mechanotransduction.

**Methods:**

Using epithelial cells transfected with inducible p60AmotL2, we employed a phenotypic drug screening approach to find compounds that specifically target invasive cells. The phenotypic screen was performed by treating cells for 72 h with a library of compounds with known antitumor activities in a dose-dependent manner. After assessing cell viability using CellTiter-Glo, drug sensitivity scores for each compound were calculated. Candidate hit compounds with a higher drug sensitivity score for p60AmotL2-expressing cells were then validated on lung and colon cell models, both in 2D and in 3D, and on colon cancer patient-derived organoids. Nascent RNA sequencing was performed after BET inhibition to analyse BET-dependent pathways in p60AmotL2-expressing cells.

**Results:**

We identified 60 compounds that selectively targeted p60AmotL2-expressing cells. Intriguingly, these compounds were classified into two major categories: Epidermal Growth Factor Receptor (EGFR) inhibitors and Bromodomain and Extra-Terminal motif (BET) inhibitors. The latter consistently demonstrated antitumor activity in human cancer cell models, as well as in organoids derived from colon cancer patients. BET inhibition led to a shift towards the upregulation of pro-apoptotic pathways specifically in p60AmotL2-expressing cells.

**Conclusions:**

BET inhibitors specifically target p60AmotL2-expressing invasive cancer cells, likely by exploiting differences in chromatin accessibility, leading to cell death. Additionally, our findings support the use of this phenotypic strategy to discover novel compounds that can exploit vulnerabilities and specifically target invasive cancer cells.

**Supplementary Information:**

The online version contains supplementary material available at 10.1186/s13046-024-03031-w.

## Background

The leading cause of death in cancer patients is typically the spread of the disease, known as metastasis, to other organs or tissues in the body [1, 2]. Metastasized cancer cells can invade other organs, disrupting their normal function, leading to organ failure and, ultimately, death. The complexity of invasive cancer and the challenges associated with treating it necessitate a comprehensive approach, potentially involving a combination of surgery, radiation therapy, chemotherapy, targeted therapy, immunotherapy, early detection, and prevention strategies [3, 4].

Despite employing a multimodal approach, treating invasive cancer remains difficult due to several factors. First, invasive cancers are often heterogeneous, containing cells with varying genetic mutations, growth rates, and treatment sensitivities, making it challenging to develop treatments that effectively target all cancer cell subpopulations [5, 6]. Second, cancer cells often develop resistance to treatments over time, especially chemotherapy and radiation therapy, either as a result of mutations or adaptations that render cells more resistant, such as the upregulation of multidrug resistant proteins, or by the presence of a small population of inherently resistant cells [7]. Additionally, the tumor microenvironment plays a crucial role in cancer growth and progression [8]. Tumor cells interact with surrounding cells, such as fibroblasts and blood vessels, promoting their growth and survival. In terms of blood vessels, tumor vessels are frequently less efficient in transporting systemic therapies into the tumor bed, hindering the drug response [9].

Further research is needed to develop targeted approaches that selectively combat invasive cancer cells and enhance the efficacy of current therapies, ultimately improving patient outcomes.

A family of proteins that has recently come under scrutiny for their role in tumor development are angiomotins, namely Angiomotin-like protein 2, or AmotL2 [10, 11]. AmotL2 has two isoforms, p100 and p60, with the p100 isoform forming a complex with E or VE-cadherin via p120catenin binding [12–16]. This complex initiates the formation of specific radial actin filaments connecting cellular junctions to the nuclear lamina [15, 17]. AmotL2 has been implicated in various biological processes, including aortic development, blastocyst hatching, epithelial cell polarity, and regulation of the Hippo signaling pathway, which controls organ size and cell growth [12, 17–20].

Moreover, it has been associated with cancer progression, metastasis, and the development of cardiovascular diseases [11, 15]. The shorter isoform, p60AmotL2, lacks the N-terminal WW protein interaction motifs and acts as a dominant negative of the full-length p100 isoform [14]. p60AmotL2 is not expressed under normoxic conditions but is activated by ischemia and found in different invasive cancers. Its expression promotes invasion, as demonstrated in both in vivo mouse tumor models and in vitro studies [13]. Interestingly, in vitro, p60AmotL2 dissociates cell‒cell contacts and thereby triggers cell invasion in an amoeboid manner [14]. These findings indicate that p60AmotL2 exerts a significant influence on the invasive behavior of cancer cells, thus underscoring its potential as a promising therapeutic target.

However, the feasibility of direct intervention using small molecular compounds presents a considerable challenge, as p60AmotL2 does not possess the structural characteristics that make it amenable to standard drug development strategies. To overcome this issue, we employed phenotypic drug screening as an approach to identify compounds that preferentially target p60AmotL2-expressing cells. Using a library of 528 previously characterized oncology drugs, we identified two classes of drugs with activity in this assay. This information sheds light on the signaling pathways critical for p60AmotL2-mediated invasion and serves as a proof-of-concept for further drug screening to discover novel anticancer compounds.

## Materials and methods

### Immunohistochemistry

Immunohistochemistry of AmotL2 in human tissues was performed in collaboration with http://www.proteinatlas.org. The following primary antibody was used: LDS-AmotL2 (polyclonal antibodies reactive to human AmotL2 C-terminal peptide and detecting both p60 and p100 isoforms [17]).

### Cell culture

Madin-Darby Canine Kidney (MDCK; product number PTA-6500, ATCC), A549 (product number CCL-185, ATCC) and SW480 (product number 87092801, ECACC) cells were cultured in Dulbecco's Modified Eagle Medium (DMEM; product number D6429, Sigma‒Aldrich) supplemented with 10% fetal bovine serum (FBS, product number 10270106, Thermo Fisher Scientific) and penicillin/streptomycin (product number 15070063, Thermo Fisher Scientific).

MDCK cells stably expressing doxycycline-inducible p60AmotL2 were produced with the Gateway™ system (Thermo Fisher) as previously described [13]. To induce p60AmotL2 expression, doxycycline (Dox; product number D3447, Sigma‒Aldrich) was added to the culture at a final concentration of 10 ng/mL. SW480 p60AmotL2 shRNA cells were generated using lentiviral vectors as previously described [14]. A549 cells constitutively expressing p60AmotL2 and respective controls were established by lentiviral infection for 3 days followed by puromycin selection (2 µg/mL, product number A1113803, Thermo Fisher Scientific). Lentiviral vectors constitutively expressing p60AmotL2 were designed by VectorBuilder using vectors coding for BC011454 (mRNA) or AAH11454.1 (protein). The exact sequence used for p60AmotL2 expression was optimized by VectorBuilder (Vector ID VB210610-1214uwv) and can be provided upon request.

### RNA-seq analysis

For RNA-sequencing, samples from the MDCK WT control (− Dox) group, MDCK WT control (+ Dox) group, MDCK p60AmotL2 control (− Dox) group and MDCK p60AmotL2 (+ Dox) group were collected. Each group consisted of quadruplicate samples to ensure data accuracy. The cells were seeded on 6-well plates and treated with or without doxycycline (10 ng/mL) for 24 h or 48 h and harvested using a Qiagen RNA extraction kit (Qiagen). All samples were processed using an RNA-seq pipeline by Novogene Co., Ltd. (Beijing, China), and RNA integrity was assessed using the RNA Nano 6000 Assay Kit on a Bioanalyzer 2100 system (Agilent Technologies, CA, USA). Subsequently, the resulting count files were employed as inputs for the DESeq2 package within the R environment. This package utilized a negative binomial distribution model to analyse differentially-expressed genes (DEGs). To enhance precision, P values were adjusted using the Benjamini and Hochberg approach. DEGs were identified based on an adjusted P value of less than 0.05 (q-value) and a log fold change greater than 0.5. Gene Ontology (GO) and Kyoto Encyclopedia of Genes and Genomes (KEGG) enrichment analyses were conducted using the clusterProfiler (v3.16.1) R package. Visual representations including volcano plots and bar plots were generated using the ggplot2 and the Enhanced Volcano R package.

### Proteomic analysis

MDCK cells were lysed with 4% SDS lysis buffer and prepared for mass spectrometry analysis using a modified version of the SP3 protein cleanup and digestion protocol [21]. Peptides were labelled with TMT10-plex reagent according to the manufacturer’s protocol (Thermo Scientific) and separated by immobilized pH gradient—isoelectric focusing (IPG-IEF) on 3–10 strips as described previously [22]. Extracted peptide fractions from the IPG-IEF were separated using an online 3000 RSLCnano system coupled to a Thermo Scientific Q Exactive-HF. MSGF + and Percolator in the Galaxy platform were used to match MS spectra to the Ensembl_92 *Homo sapiens* protein database [23].

### Western blotting (WB)

To prepare whole-cell lysates, cells were treated with a lysis buffer that contained 50 mM HEPES buffer, 150 mM NaCl, 1.5 mM MgCl_2_, 1 mM EGTA, 10% glycerol, and 1% Triton X-100 (product number X100, Sigma‒Aldrich), along with a freshly added 1 × protease inhibitor (product number 04693159001, Roche). This process was performed on ice. Next, the mixture was subjected to centrifugation at 15,000 rpm for a span of 3 min and the supernatant was collected. Lysates were then combined with SDS sample buffer (4X, product number 1225644, Novex) that was enhanced with a 10% sample reducing agent (product number 1176192, Novex). The proteins present were fractionated using a Bis–Tris precast polyacrylamide gel with a gradient of 4–12% (product number NP0322BOX, Novex). These fractionated proteins were then transferred onto a nitrocellulose membrane (product number 10401396, Whatman). To block any non-specific binding, the membrane was treated with a solution of 5% nonfat dry milk and 0.1% Tween 20 in phosphate-buffered saline (PBS) for an hour at room temperature. Following this, the membrane was incubated overnight at 4 °C with a primary antibody and was then subjected to treatment with a secondary antibody for an additional hour at room temperature. Finally, proteins that were labelled with antibodies were identified using a chemiluminescent substrate (ECL; product number RPN2232, Amersham) on an iBright imaging system (Thermo Fisher Scientific, USA). For primary antibodies we used LDS-AmotL2 (in-house polyclonal antibodies reactive to human AmotL2 C-terminal peptide and detecting both p60 and p100AmotL2 isoforms), β-actin (product number ab3280, Abcam) and Vinculin (product number 13901, Cell Signaling Technology). For secondary antibodies we used ECL anti-mouse IgG horseradish peroxidase (product number NA931V, Cytiva) and ECL anti-rabbit IgG horseradish peroxidase (product number NA934V, Cytiva).

### Drug sensitivity and resistance testing

Cell lines were seeded using a Multidrop dispenser (Thermo Fisher Scientific) into pre-spotted 384-well plates (product number 6007668, PerkinElmer) containing 528 oncology compounds in five concentrations (FIMM oncology collection, FIMM High-Throughput Biomedicine Unit) or a custom drug library made with an Echo 550 (Beckman Coulter) [24]. MDCK cells were added at a final concentration of 2000 cells/well. Doxycycline was added to the cell culture media prior to dispensing. The drug plates were incubated for 72 h at 37 °C, after which cell viability was determined by adding CellTiterGlo (CTG; product number G7573, Promega) and measuring the luminescence signal with an EnSight plate reader (PerkinElmer). Dose response curves and drug sensitivity scores (DSS) were calculated with an in-house analysis pipeline termed Breeze [25]. Briefly, dimethyl sulfoxide (DMSO) and benzethonium chloride (BzCl) were used as negative and positive controls, respectively, to calculate the dynamic range of the assay for each plate, which was then used to calculate DSS values for each compound [26]. An initial primary screen with the 528 compounds tested over 5 different doses was performed. Selective drug sensitivity scores (sDSS) were used to quantify the selective response of p60AmotL2-expressing cells relative to non-expressing control cells. This was done by subtracting the DSS value for control cells from the DSS value of p60AmotL2-expressing cells for each compound [26]. The top 60 compounds in terms of sDSS values were selected for a second round of screening, where 9 doses per compound were used to make the dose‒response profiles instead of the initial 5. For these studies, custom plates were created from the Nordic Oncology Set by the Compound Center at Chemical Biology Consortium Sweden (CBCS), and the same in-house analysis pipeline was used to format the data for analysis in Breeze [25]. New DSS and sDSS values were calculated based on these results, and the compounds from the two top classes identified across both the primary screen and the validation screen were used for further validation.

### Fluorescence staining of nuclei in 2D

Briefly, MDCK cells were seeded on 96-well plates (product number SW96G-EC-HTS, Cell Guidance Systems) at a density of 10 000 cells per well. Once cells reached approximately 60 to 70 percent confluency (approximately 2 days), compounds (BETi and RTKi) were added at different concentrations. After 72 h treatment, the cells were washed two times with 1 × PBS and fixed with 4% paraformaldehyde (PFA; product number sc-281692, Santa Cruz Biotechnology) for 10 min at room temperature. After two more rounds of washing with 1 × PBS, cells were incubated for 30 min at room temperature with NucBlue™ Live ReadyProbes™ Reagent (product number R37605, Thermo Fisher Scientific), diluted in 1 × PBS according to the manufacturer’s instructions, to stain the nuclei. Plates were imaged using a Leica Thunder Wide Field Fluorescence Microscope (Leica, Germany).

### Validation of hit compounds in 2D and 3D using CellTiterGlo

For 2D cell viability testing, cells were seeded in 384-well plates (product number 6007668, PerkinElmer) at a density of 1000 cells per well in 20 µL growth media. Compounds were added the following day in 10 µL growth media and incubated for 72 h. DMSO and BzCl were used as negative and positive controls, respectively. Cell viability was determined by adding 30 µL of CellTiterGlo (CTG; product number G7573, Promega) and incubating for 15 min while protected from light. 3D viability testing was performed in parallel from the same cell stocks. We seeded approximately 500 cells per well in a 20 µL Geltrex (product number A1413202, Thermo Fisher Scientific) plus 10 µL growth media mix. Organoids were allowed to grow for 3 to 4 days before adding compounds in 10uL growth media for 72 h. The exception was SW480 cells, since these were grown in a collagen matrix instead of Geltrex, using the same seeding protocol as the one used for MDCK cells detailed in the method section below pertaining to the Immunofluorescence Staining of 3D Collagen Gels. We then added 40 µL of CellTiter-Glo® 3D Cell Viability Assay (product number G9681, Promega) and incubated for 30 min with gentle agitation, protected from light. The luminescence signals were measured with a Varioskan LUX microplate reader (Thermo Fisher Scientific, USA). The dose‒response curves and relative IC50 values were generated by using a dose‒response nonlinear regression model from GraphPad Prism (Dotmatics, USA).

### Immunofluorescence staining of 3D collagen gels

Cells were seeded on 8-well chamber slides (product number 154534, Thermo Fisher Scientific) in a collagen matrix (PureCol type I collagen; product number 5005, Advanced Biomatrix) at a density of 500 cells per well. Compounds were added at different concentrations after five to seven days in culture. After 48 h of treatment, collagen gels underwent two washes in 1 × PBS and were then fixed in 0.5 mL of 4% paraformaldehyde (PFA; product number sc-281692, Santa Cruz Biotechnology) for 30 min. Afterwards, the gels were washed three times with 1 × PBS and permeabilized using 0.1% Triton X-100 (product number X100, Sigma‒Aldrich) for a duration of 15 min. After an additional two washes in 1 × PBS, the primary antibody, diluted in 5% normal horse serum, was added and allowed to incubate at 4 °C overnight. The gels were subsequently washed three more times in 1 × PBS and then incubated with the secondary antibody for 1.5 h at room temperature. Following another three washes in 1 × PBS, the gels were prepared for imaging by removing the chambers from the slides and using Fluoroshield™ with DAPI (product number F6057, Sigma‒Aldrich) to carefully mount the gels between the slides and coverslips. Cleaved Caspase-3 (product number 9661, Cell Signaling Technology) was used as the primary antibody and Alexa Fluor 488 anti-rabbit (product number A21441, Thermo Fisher Scientific) was used as the secondary antibody. Gels were also stained with Phalloidin-Atto 647 N (product number 65906, Sigma‒Aldrich) to visualize actin filaments. Confocal images were captured using an LSM 700 microscope (Zeiss, Germany) and all image processing was carried out using ImageJ software.

### Patient-derived colon organoid cultures

#### Isolation and culture of colon organoids

Fresh samples from surgically resected colon tumor specimens and paired healthy colon tissues were obtained from the Department of Clinical Pathology and Cancer Diagnostics at Karolinska University Hospital, Stockholm, Sweden. Experimental procedures and protocols were approved by the regional ethics review board (Etikprövningsnämnden) in Stockholm. Clinical information for each patient specimen involved in this study is listed in Supplemental data 6. The tissue samples were washed with ice-cold PBS containing 1% penicillin–streptomycin (Gibco) repeatedly until the solution became clear. The tissues were then minced into small fragments (1–2 mm) and incubated with Gentle Cell Dissociation Reagent (product number 100-0485, Stemcell Technologies) on a shaker (~ 20 rpm) for 15 min followed by centrifugation. After carefully removing the supernatant, the remaining tissues in the bottom were resuspended in cold PBS with 1% BSA and filtered through a 70 μm cell strainer to remove undigested tissue debris. The processed suspension was centrifuged, and the resulting pellet was mixed with Geltrex (product number A1413202, Thermo Fisher Scientific) at a 1:1 ratio. 50 µl of Geltrex-organoid mixture was then plated in pre-warmed 24-well plates and allowed to solidify at 37 °C. IntestiCult™ Organoid Growth Medium (Human) (product number 06010, Stemcell Technologies) with Y-27632 (product number 72302, Stemcell Technologies) was gently added to the wells. The organoids were cultured in a humidified incubator at 37 °C with 5% CO_2_ and the medium was changed every 3 days.

### Lentiviral infection and generation of stable PDO models

A third-generation lentiviral transduction system was used to establish stable p60AmotL2-expressing organoids. p60AmotL2 and the respective control plasmids were co-transfected with plasmids expressing virus coat and assembly proteins (REV, RRE, and VSVG) into 70% -80% confluent HEK293T cells using Lipofectamine 3000 (product number L3000001, Invitrogen). Conditioned media were collected after 24, 48 and 72 h, filtered through 0.45 μm low protein binding membranes (Sarstedt) and later concentrated at 3200 xg for 15 min. The supernatant media containing virus particles were then concentrated using a PEG Virus Precipitation Kit (product number ab102538, Abcam) and stored at $-$ 80 °C for future use. Colon organoids were dissociated into small clusters using Gentle Cell Dissociation Reagent (product number 100-0485, Stemcell Technologies)﻿ and resuspended in culture medium with concentrated lentiviral particles. The organoid suspension was then mixed with Geltrex (product number A1413202, Thermo Fisher Scientific) and plated in a 24-well plate. The growth media was replaced every other day. Protein expression was examined by western blotting and fluorescence microscopy. The selected organoids were expanded by passaging and maintained in culture for further experimentation.

### Drug screening using stable PDO models and cell lines in 3D

Stable PDO models were plated in 384-well plates (product number 6007668, PerkinElmer) at approximately 500 cells per well in a 20 µL Geltrex (product number A1413202, Thermo Fisher Scientific) plus 10 µL growth media mix. Organoids were allowed to grow for 3 to 4 days before adding compounds in 10 µL growth media for 72 h. We then added 40 µL of CellTiter-Glo® 3D Cell Viability Assay (product number G9681, Promega) and incubated for 30 min with gentle agitation, protected from light. The luminescence signals were measured with a Varioskan LUX microplate reader (Thermo Fisher Scientific, USA). The dose‒response curves and relative IC50 values were generated by using a dose‒response nonlinear regression model from GraphPad Prism (Dotmatics, USA).

### Nascent RNA sequencing using Global Run-on sequencing (GRO-seq)

#### Pulse labelling of RNA

MDCK cells were seeded in 10 cm dishes at a confluency of 500 000 cells per dish. After 48 h, Dox was added overnight to the culture at a final concentration of 10 ng/mL to induce p60AmotL2 expression. Cells were then treated with 5 μM iBET151 or DMSO vehicle control for 3 h, after which 0.5 mM (final concentration) 5’-ethynyl uridine (EU; Click-it Nascent RNA Capture kit, product number C10365, Invitrogen) was added for 30 min to the cells to label newly synthesized (nascent) RNA. Cells were then washed briefly for three times with 1 × PBS and, after detachment with trypsin, pelleted for 5 min at 500 xG and lysed with Cell Disruption Buffer (product number AM1921, Invitrogen) for 5 min on ice. Total RNA was extracted using PARIS kit (product number AM1921, Invitrogen) according to the manufacturer’s instructions.

### Streptavidin pulldown of labelled RNA

The Click-it reaction to biotinylate the labelled RNA was performed according to manufacturer’s instructions with minor modifications (Click-it Nascent RNA Capture kit (product number C10365, Invitrogen). Briefly, 1000 ng of EU-labelled RNA was added to a buffer containing CuSO_4_ and biotin azide for biotinylation of the ethynyl group. The reaction started with addition of Additive 1 from the kit and took place for 3 min before being ceased by adding Additive 2. After 30 min of incubation in a rotator at RT, biotinylated RNA was precipitated overnight in a solution of ethanol and LiCl_2_ in a -80 °C ultrafreezer. After isolation (20 min centrifugation at 14,000 RPM, followed by two washes in 70% ethanol), biotinylated RNA was purified using streptavidin beads provided by the kit. Incubation of 60 min at RT was followed by ten washes as instructed in the user’s guide, and the captured RNA adhered to the beads was used for library preparation. As a control, click-it reaction and pull down was performed on unlabelled RNA isolated from MDCK cells with no EU treatment.

### Library generation and sequencing

Lexogen’s QuantSeq 3’ mRNA library preparation kit FWD for Illumina (product number 015, Lexogen) with i7 indices was used for library preparation following the manufacturer’s protocol for low input RNA samples, with minor modifications. Briefly, the 5 μl of resuspended beads with 1 µl of diluted (1/10000) ERCC ExFold RNA Spike-In Mix (product number 4456740, Thermo Fisher Scientific) was used as starting material for the first strand synthesis reaction, followed by RNA removal and second strand synthesis reaction according to the manufacturer’s instruction. The converted dsDNA library was carefully removed from the beads by using a magnetic rack. These libraries were further purified using purification beads provided with the kit, followed by library amplification with i7 indices and repurification. The quality and fragmentation of purified libraries was assessed on a Bioanalyzer (Agilent Technologies, USA). Sequencing was performed on Novogene’s (Cambridge, United Kingdom) sequencing platform Novaseq 6000 (Flowcell S4, software version V1.7, reagent V1.5) using a Pair-end 150 sequencing strategy (50 M read depth).

### Data processing

Data analysis was performed by Lexogen’s proprietary Data Analysis Pipeline (DAP) with default parameters for QuantSeq (https://www.lexogen.com/quantseq-data-analysis/). In brief, adapters and low-quality reads were filtered by Cutadapt (1.18) (https://cutadapt.readthedocs.io/en/stable/). Differential expression analysis on gene counts based on full gene coordinates and exon coordinates was performed using DESeq2 (1.18.1) [27] and Canis lupus familiaris Ensembl 107 (https://ftp.ensembl.org/pub/release-107/fasta/canis_lupus_familiaris/, https://ftp.ensembl.org/pub/release-107/gtf/canis_lupus_familiaris/) as a reference genome.

For this, clean reads were aligned to the reference genome and ERCC RNA spike-in fasta (Thermo Fisher Scientific) with STAR aligner (2.6.1a) (https://github.com/alexdobin/STAR) [28] using custom parameters summarized in the table as follows:

**Table:** 

Parameter	Value
outFilterMismatchNoverLmax	0.6
alignSJoverhangMin	8
alignEndsType	Local
outFilterMultimapNmax	200
alignSJDBoverhangMin	1
outFilterMismatchNmax	999
alignIntronMin	20
alignIntronMax	1,000,000
alignMatesGapMax	1,000,000
limitOutSJcollapsed	5,000,000

Gene counts from uniquely aligned reads were obtained using subread’s featureCounts (1.6.4) (https://subread.sourceforge.net/). Significant genes identified based on exon feature unique counts from differential gene analysis were proceeded for Gene ontology (GO) enrichment analysis. Assessment for all relations such as Molecular Function, Cellular Component, Biological Process (< 0.05 p-adj, BH) was performed using ClusterProfiler R package (v4.10.0) [29], universe baseMean > 1. For the Gene Set Enrichment Analysis (GSEA) and the pathway analysis msigdbr (7.5.1) and ReactomePA (1.46.0) R packages were used, respectively.

## Results

### AmotL2 expression in invasive cancers

**Fig. 1:**
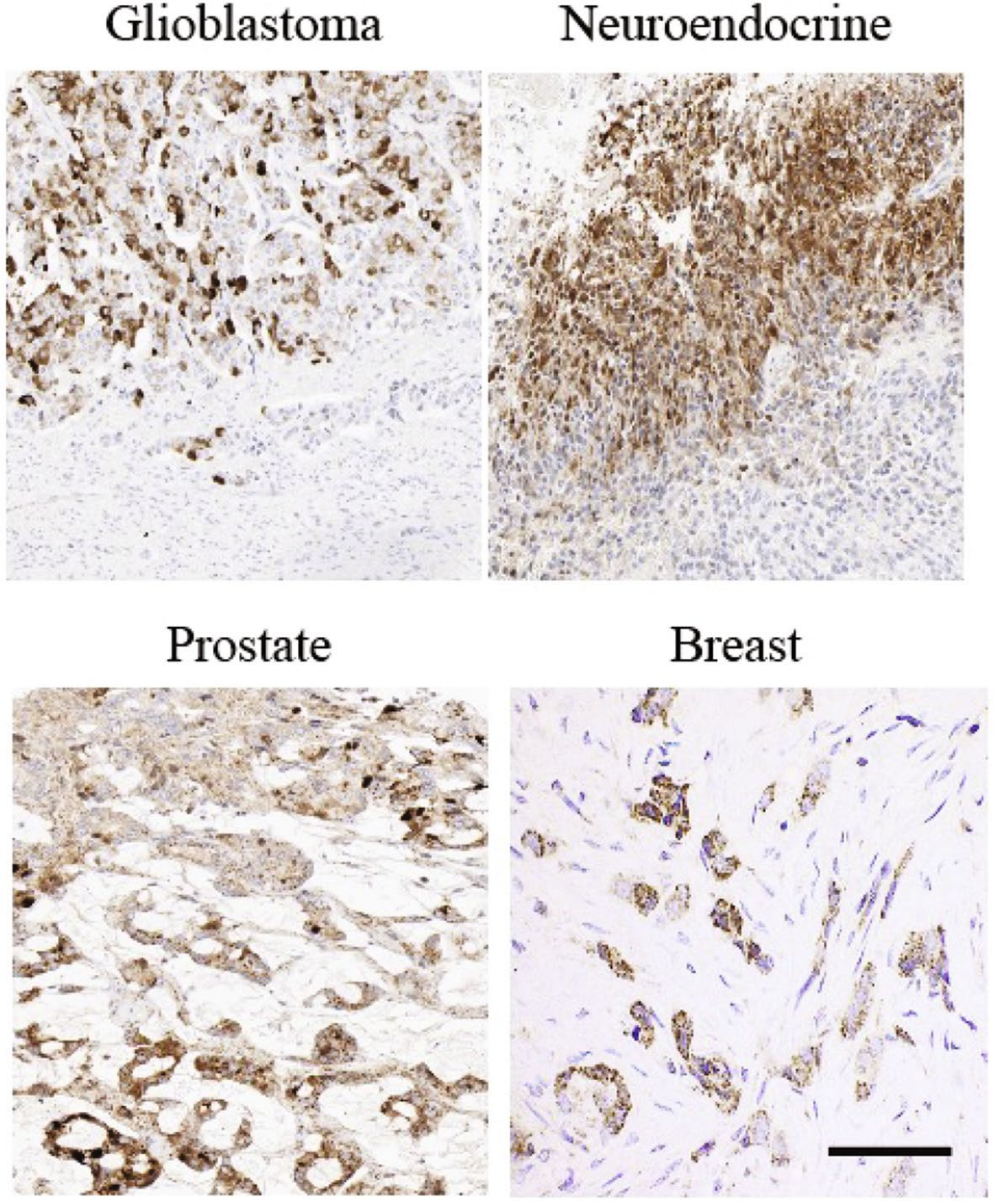
AmotL2 expression in invasive cancers. Immunohistochemical analysis of AmotL2 expression in human glioblastoma, neuroendocrine, prostate and breast cancer. Note the cytoplasmic vesicular staining in brown, characteristic of p60AmotL2 expression. Scale bar = 50 μm

In a collaborative effort with the human protein atlas consortium (HPA), we conducted an analysis of AmotL2 expression across various tumor types. The antibodies utilized in this study were developed by the Holmgren group, and the subsequent staining procedures were carried out by the HPA consortium. These antibodies detect both the p100 and p60AmotL2 isoforms, with the former showing a characteristic staining localized to the cellular junctions and the latter displaying cytoplasmic vesicular staining. We have previously shown that p60AmotL2 is expressed in metastatic colon cancer [13, 16] . The immunostainings in Fig. 1 show positivity in the invasive fronts of several other tumor types such as glioblastoma, neuroendocrine, prostate and breast cancers.

To understand the molecular mechanisms underlying the migratory plasticity of tumor cells, we used a doxycycline (Dox)-inducible p60Amotl2-expressing Madin-Darby Canine Kidney (MDCK) cell model to perform systematic molecular profiling. This cell line was chosen as it is the gold-standard for epithelial studies in vitro [30, 31], and we have extensively documented the effect of p60AmotL2 in these cells [13, 14, 16]. Although MDCK cells exhibit a robust epithelial phenotype, these are non-transformed cells, so we wanted to elucidate whether this model would be suitable for phenotypic drug screening.

**Fig. 2:**
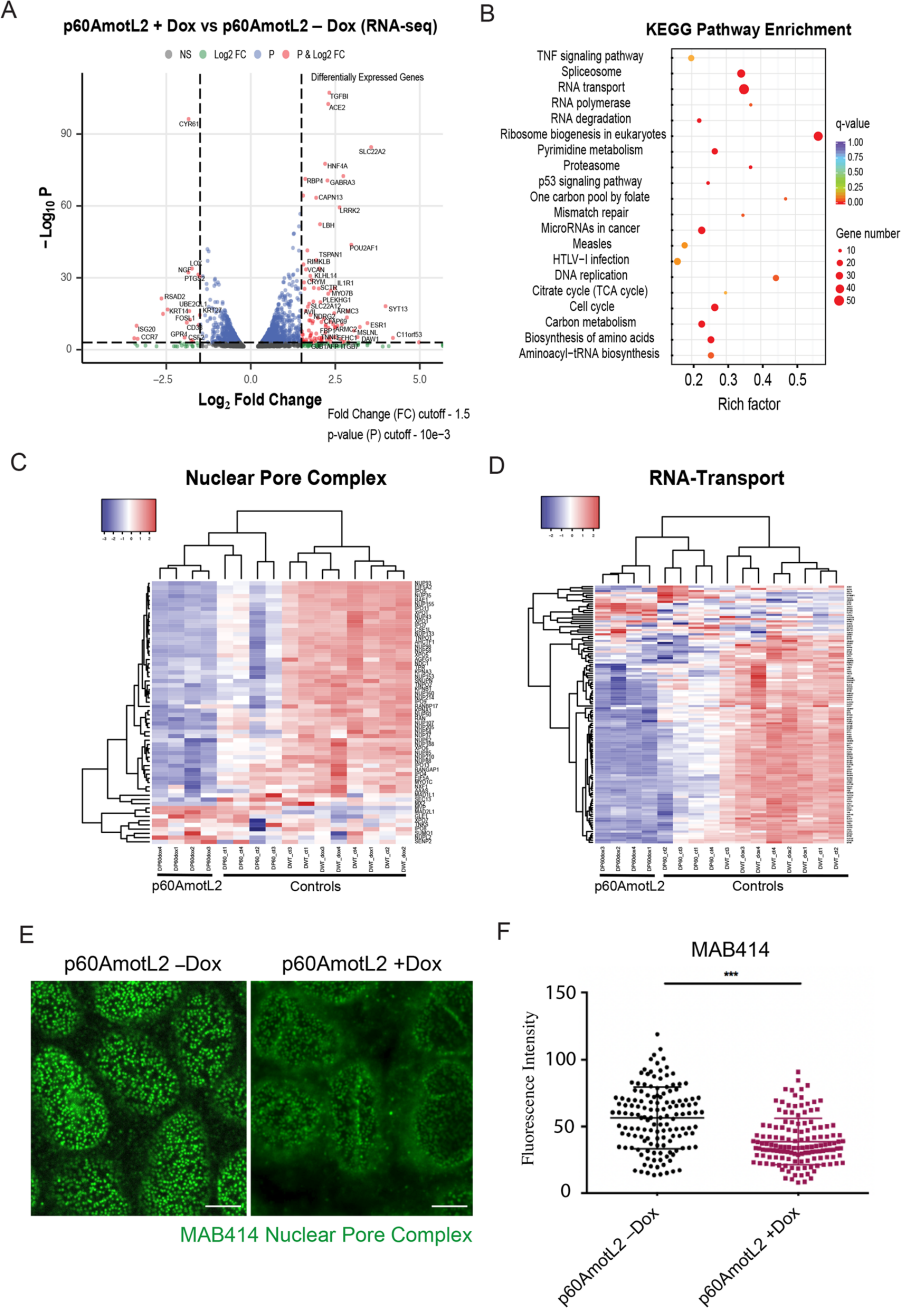
RNA-seq analysis of p60AmotL2-induced gene expression in MDCK cells. RNA expression was analysed in MDCK cells after Dox induction of p60AmotL2 expression. To exclude the influence of Dox on gene expression, Dox-treated control cells were analysed in parallel (Supplemental Data 1). **A** Volcano plot to visualize the results of differential expression between p60AmotL2 $+$ and p60AmotL2 $-$ cells. Label description: NS – non significant; FC – fold change; P – *p*-value. **B** Identification of KEGG pathways affected by p60AmotL2 expression. The size of each circle represents the number of transcripts involved in the corresponding pathway, and the color scale is related to the q-value. **C** and **D** RNA-seq heatmap analysis provides a visual overview of KEGG pathways identified in B. **E** Immunofluorescence staining using MAB414 monoclonal antibody, which recognizes a family of nuclear pore complex (NPC) proteins. Note the decreased fluorescent intensity of nuclear pore-associated MAB414 antibodies in p60AmotL2-expressing cells. **F** Scatter plot showing quantification of the immunofluorescence signal of MAB414 in the indicated conditions (n = 142, corresponding to individual nuclei; Student’s T test, *** *p* < 0.001). Data represent the mean ± SD from three independent experiments. Scale bar = 5 μm

To this end, we performed transcriptomic analysis of control and p60AmotL2-expressing MDCK cells. Interestingly, relatively few genes were found to be differentially expressed (Fig. 2A and Supplemental data 1). However, consistent with previously observed changes in the nuclear membrane of p60AmotL2-expressing cells [14], we found that genes encoding nuclear pore proteins, as well as nuclear transport proteins, were significantly down-regulated (Fig. 2C). Our RNA-seq data suggested changes in the integrity of the nuclear membrane that may affect mRNA transport between the nuclear and cytoplasmic compartments (Fig. 2D).

While changes in mRNA expression were observed, they did not provide clear insights into the regulation of the pro-invasive phenotype found in p60AmotL2-expressing cells (Fig. 2B). This suggested that regulation may occur at the post-translational level instead. Therefore, we performed protein profiling using the proteomics platform at SciLifeLab, Stockholm.

**Fig. 3:**
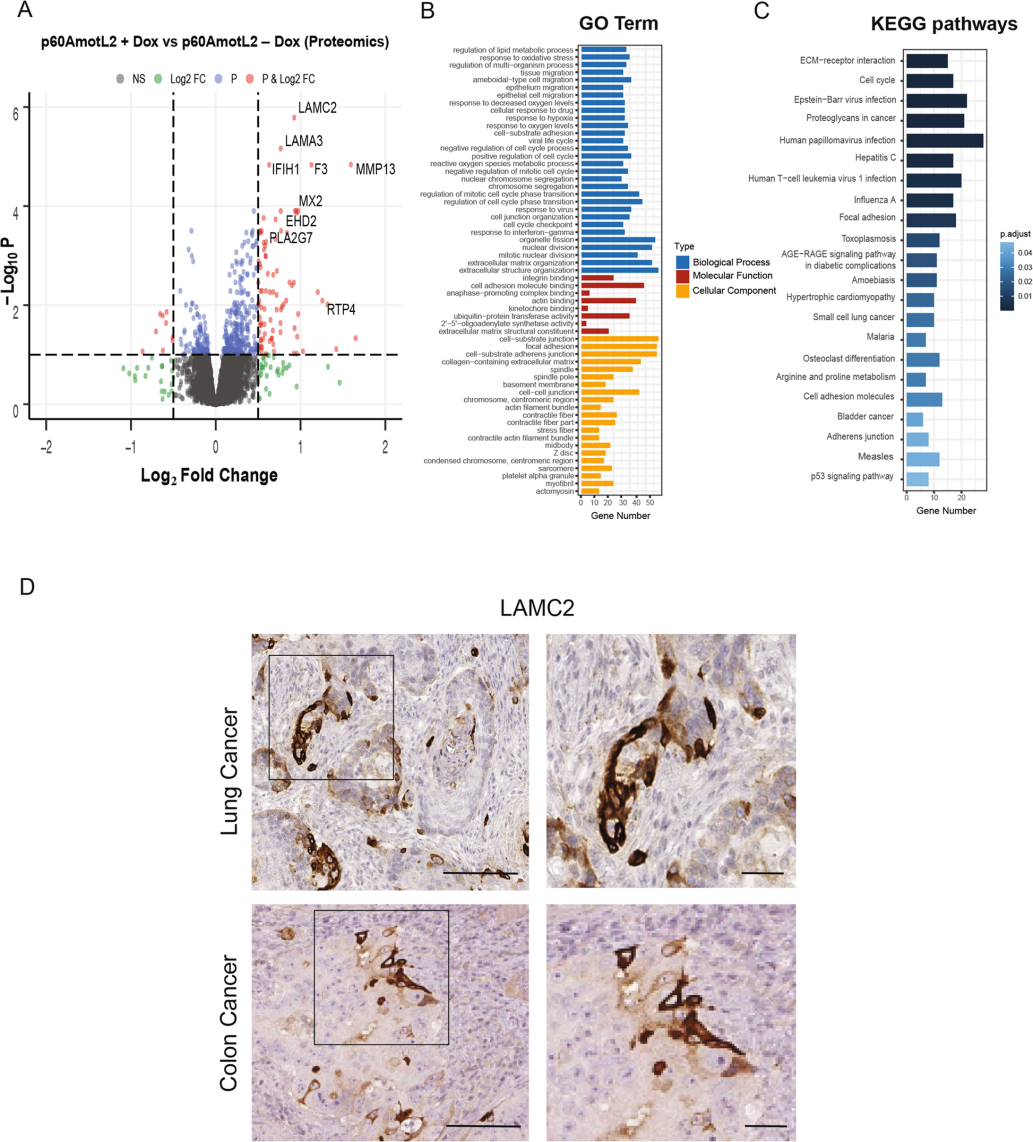
A comprehensive proteomic analysis of proteins affected by p60AmotL2 expression. **A** Volcano plot showing the individual changes in 9,136 proteins in epithelial cells after induction of p60AmotL2. Different colors indicate filtering criteria; red represents proteins with a Log_2_ fold change > 0.5 or < -0.5 and Log_10_ P value < 0.05. Proteomics data can be found in Supplemental data 2. **B** Identified proteins were annotated by Gene Ontology (GO). **C** KEGG analysis of the identified proteins. **D** Immunohistochemical (IHC) staining of LAMC2 in lung cancer (upper panel) and colon cancer (lower panel). Images on the right represent insets shown as black squares in the images on the left. Left scale bar = 100 μm, right scale bar = 25 μm

The analysis showed the relative levels of over 9 000 proteins covering most of the epithelial proteome. Interestingly, we found that p60AmotL2 expression induced a specific set of pro-invasive proteins/protein pathways, as shown in Fig. 3A, B and C (list of differentially-expressed proteins presented in Supplemental data 2). These include the alpha2, beta3 and gamma2 chains that together form Laminin-5. Laminin-5 is a major component of the extracellular matrix (ECM) and is involved in cell adhesion and migration. In cancer, Laminin-5 has been found to promote tumor cell invasion by facilitating the detachment of cancer cells from the primary tumor and their subsequent migration and invasion of adjacent tissues (Fig. 3D) [32, 33]. Other positive hits included MMP13, which is one of three genes recently identified as being drivers of lung cancer brain metastasis [34]. Tissue Factor (TF), which is involved in tumor angiogenesis and invasion, was also identified [35].

Overall, while changes in mRNA expression did not offer clear insights into the regulation of the pro-invasive phenotype, protein profiling revealed that p60AmotL2 activation induced a distinct set of pro-invasive proteins and pathways. This finding suggested that p60AmotL2-expressing MDCK cells could be used for invasion-linked phenotypic drug screening.

### A phenotypic drug screening approach to target p60AmotL2 expressing cells

**Fig. 4:**
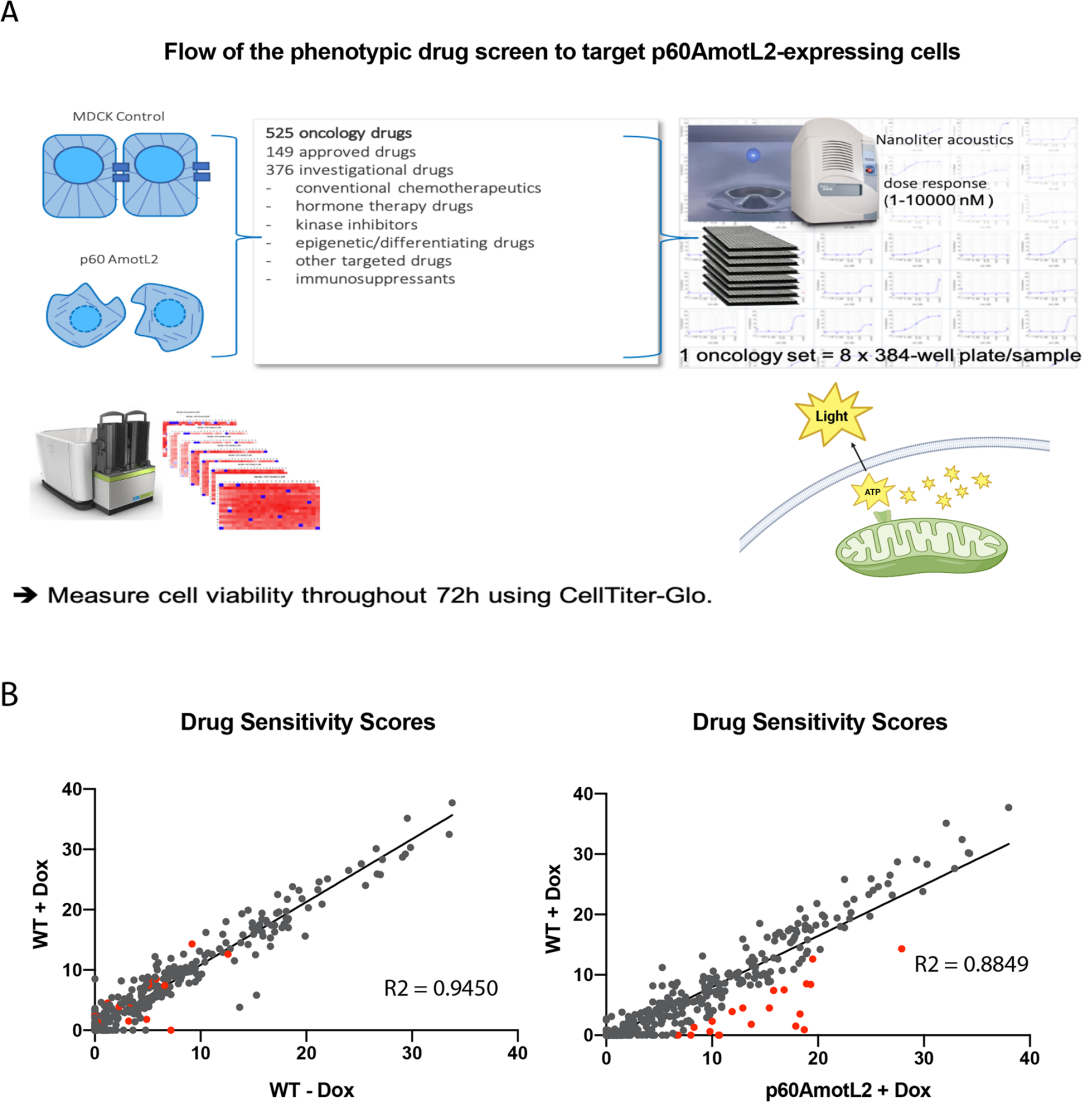
Phenotypic drug screen workflow with Drug Sensitivity Scores (DSS) for 528 oncology drugs. **A** The drugs were tested in five doses ranging from 1–10,000 nM for 72 h after which viability was measured by Cell Titer-Glo. **B** Scatter plots showing the Drug Sensitivity Scores (DSS) of individual compounds. The scatter plot on the right highlights outlier compounds (in red) with high DSS values for p60AmotL2-expressing cells, while displaying lower DSS values for control cells. On the left scatter plot, we compare DSS values for WT MDCK cells with and without Dox, accounting for Dox-related effects

Next, we investigated whether it would be possible to specifically target p60Amotl2-expressing cells. Therefore, we performed viability drug testing using a pharmacopeia-wide library of 528 established and emerging oncology drugs as previously described [24, 26, 36, 37]. We performed assay optimization to ensure optimal cell density and correct cell‒cell contacts for the 72-h assay duration, and viability was measured by cellular ATP levels using Cell-Titer Glo (CTG) (Fig. 4A). All 528 compounds were tested in five doses spanning a 10,000-fold concentration range in a final volume of 25 μL/well on control and p60AmotL2-expressing cells.

The initial drug testing was carried out using MDCK cells. Initially, we compared Dox-inducible p60AmotL2-expressing cells with wild-type (WT) MDCK cells treated with Dox to exclude the influence of Dox-related effects (Fig. 4B). We analysed the data through a web-based pipeline that included extensive assay quality control (Supplemental data 3) measuring parameters such as Z', Strictly Standardized Mean Difference (SSMD), and visualization of potential edge effects or striping patterns that are inherent to high-throughput screening (HTS) assays [38]. The pipeline also automatically generated curve fits for all drugs and hit scoring by calculating the multi-parametric drug sensitivity score (DSS) [26].

Our initial analysis of the viability testing from the primary screen yielded approximately 60 compounds that showed increased efficacy in inhibiting the viability of p60Amotl2-positive cells, with selective drug sensitivity scores (sDSS) above 4.5 (Supplemental data 4). We then performed a secondary validation screen retesting only those 60 compounds but using nine drug concentrations per compound instead of five. Of note, these compounds were sourced from different vendors for the validation screen, lending confidence to the confirmed hits. Interestingly, a substantial portion of these compounds exhibited discernible segregation into two primary categories: receptor tyrosine kinase inhibitors (RTKi), most of which exert their effects on EGFR and c-met growth factor receptors, and BET inhibitors (BETi) (Table 1).

**Table 1:**
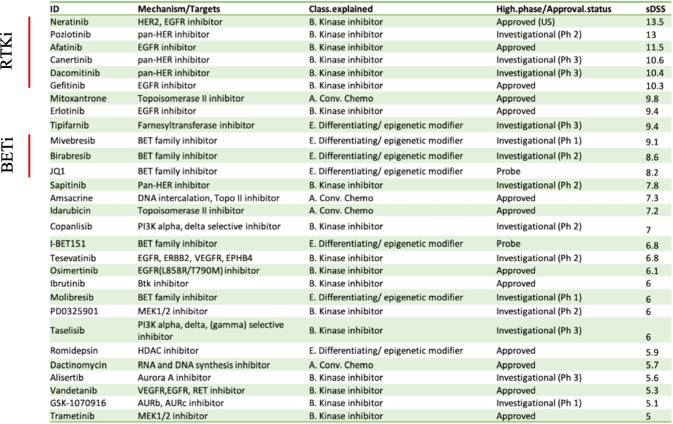
Inhibitors with higher selective Drug Sensitivity Scores (sDSS) for p60AmotL2-expressing cells compared to controls. This table highlights inhibitors that demonstrated higher sDSS for p60AmotL2-expressing cells in the validation screen, indicating drugs that selectively inhibit p60AmotL2-expressing cells compared to control cells. The sDSS was calculated by subtracting the Drug Sensitivity Score (DSS) value for control cells from the DSS value of p60AmotL2-expressing cells for each compound. sDSS values for both the primary screen and the validation screen for all 60 hit compounds are available in Supplemental Data 4

**Fig. 5:**
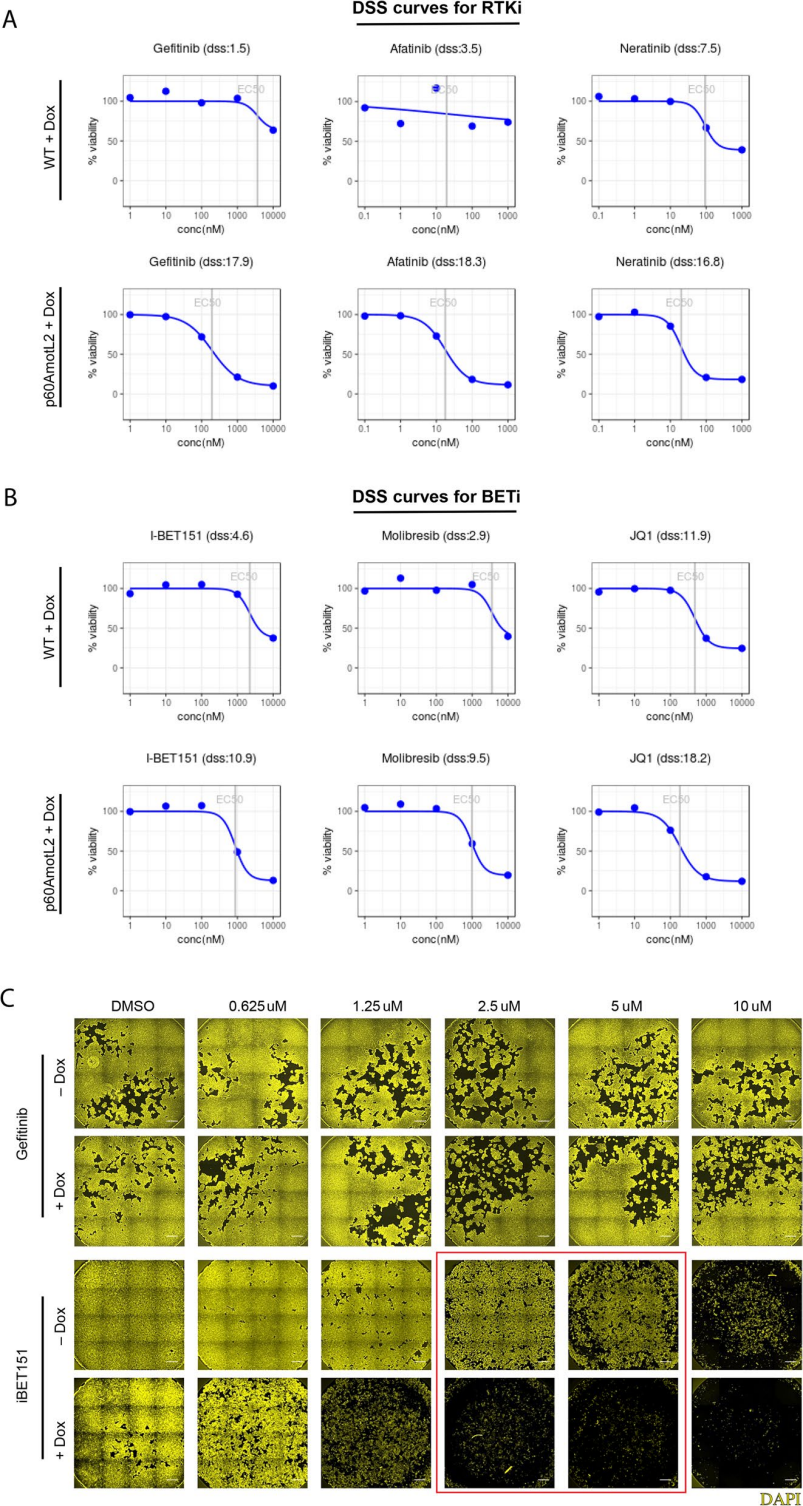
Curve fits and immunofluorescence (IF) validation for the two main classes of compounds targeting p60AmotL2-expressing cells. **A** and **B** Curve fits from the primary screen depicting the percentage of viability measured by CTG after 72 h of treatment with a 5-dose range of the respective inhibitors, RTKi (**A**) and BETi (**B**). Three representative compounds from each class are shown. The curve fits highlight the EC50 values of each inhibitor (represented by a gray line) and display the corresponding Drug Sensitivity Scores (DSS) for both p60AmotL2-expressing cells and their respective controls. **C** Immunofluorescence (IF) imaging validation in 2D after 72 h of treatment with Gefitinib (upper panel) and iBET151 (lower panel). Cell nuclei were stained with Hoechst 33342 (yellow). Red square is meant to highlight the difference in response to iBET151 of p60AmotL2-expressing cells when compared to controls. Scale bar = 500 μm

While EGFR inhibitors (EGFRi) such as Gefitinib were successful in inhibiting the metabolic viability of p60AmotL2-expressing cells in the primary and validation screens (Fig. 5A), where cells were grown for 72 h in plates pre-spotted with the respective compounds, these inhibitors failed to replicate those efficacies when the compounds were added after cells had reached over 70% confluency (Fig. 5C, EGFRi/Gefitinib top panel). On the other hand, BETi showed an ability to both inhibit the viability of p60AmotL2-expressing cells when exposed to these inhibitors from the moment of seeding (Fig. 5B) and to kill these cells when treated at approximately 70% confluency (Fig. 5C, BETi/iBET151 lower panel), when compared to controls. Notably, at concentrations of 2.5 μM and 5 μM of iBET151 (demarked by the red box in Fig. 5C), approximately 80% of MDCK p60AmotL2-expressing cells had been killed, while control cells showed little to no effect.

### Validation of anti-cancer activities of candidate compounds

**Fig. 6:**
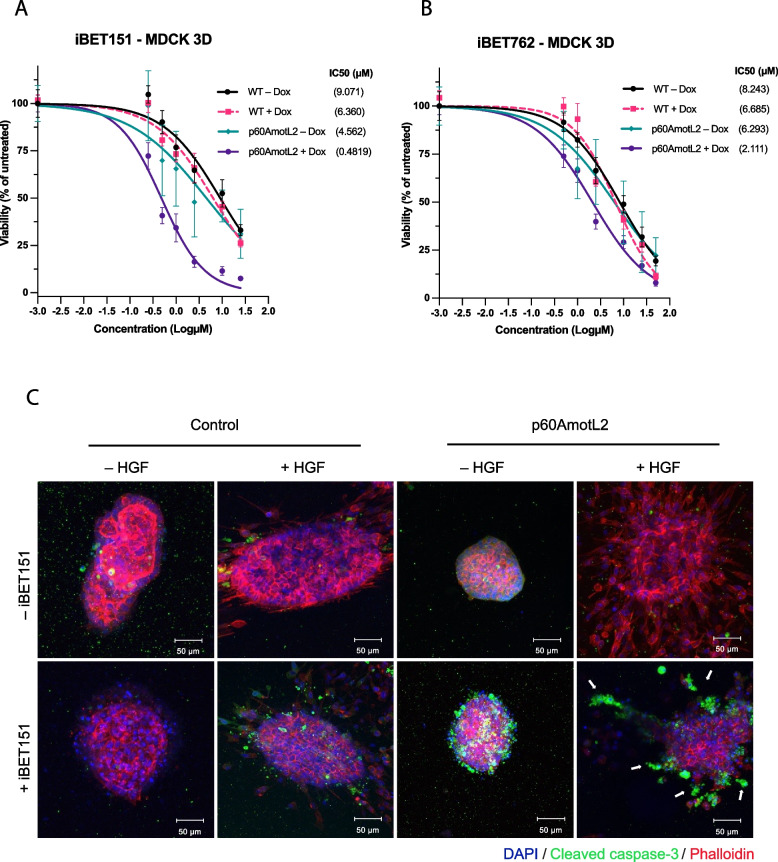
Validation of iBET151 and iBET762 on MDCK p60AmotL2-expressing cells in 3D gels. **A** and **B** Dose‒response viability testing of MDCK cells grown in 3D Matrigel with CTG after 72 h of treatment with iBET151 (**A**) and iBET762 (**B**), respectively. The IC50 values were calculated using the dose‒response nonlinear regression model from GraphPad Prism. The percentage of viability for p60AmotL2-expressing cells is represented by the purple line, and the values are compared to controls. From top to bottom, 95% CI for iBET151 (**A**): [5.992, 14.82], [4.748, 8.732], [2.950, 7.570] and [0.3647, 0.6111]; 95% CI for iBET762 (**B**): [6.073, 11.23], [5.154, 8.674], [4.626, 8.594] and [1.611, 2.748]. **C** IF confocal imaging of MDCK cells grown in 3D collagen. Cells were cultured in a collagen matrix for approximately 7 days, treated with Dox for 48 h to induce p60AmotL2 expression, and exposed to HGF for 24 h to induce migration of control cells and scattering of p60AmotL2-expressing cells. Subsequently, cells were treated with iBET151 for 24 to 48 h before fixation. Cleaved caspase-3 staining (in green) was used to assess cell inhibition or cell death; white arrows indicate migrating cells positive for cleaved caspase-3 following iBET151 treatment. Data represent the mean ± SEM from three independent experiments. Scale bars = 50 μm

The p60AmotL2-driven invasive phenotype has been extensively documented in three-dimensional (3D) models embedded within a collagen matrix, as previously reported [13, 14]. Therefore, the activity of the identified BET inhibitors was evaluated in MDCK 3D cultures. As shown in Fig. 6A and B, a significant decrease in cell viability was observed in 3D-cultured p60AmotL2-expressing cells (marked in purple) upon treatment with BETi compared to controls. The relative IC50 values between p60AmotL2 Dox-treated cells and Dox-untreated cells showed a 9.5-fold difference for iBET151 and a 3.0-fold difference for iBET762. Intriguingly, we observed that iBET151 primarily induced cell death in individual migrating cells within the collagen matrix, as shown by their positive cleaved caspase-3 immunofluorescence (IF) staining depicted in green in Fig. 6C (indicated by the white arrows in the lower right panel).

**Fig. 7:**
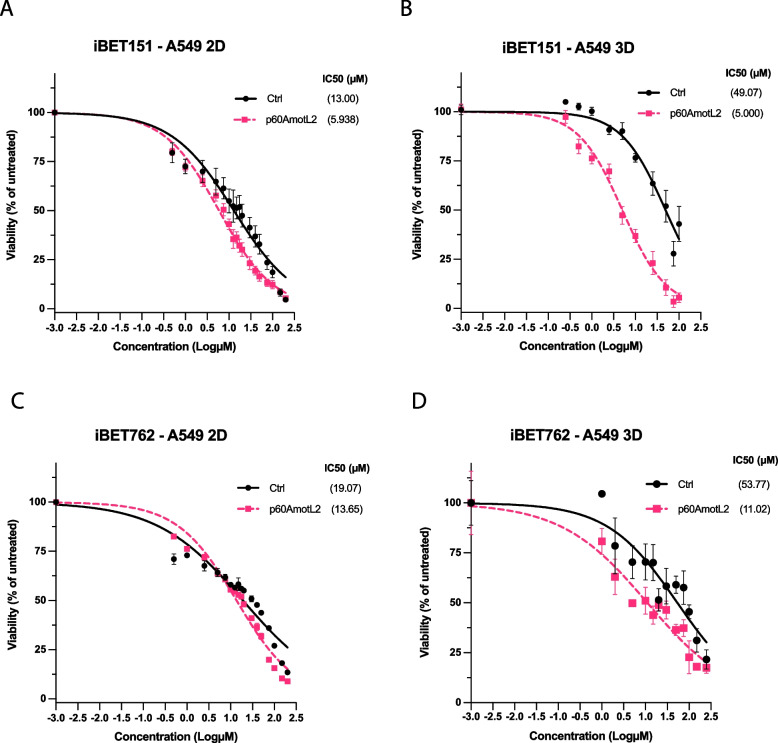
Efficacy of iBET151 on A549 p60AmotL2-expressing cells in 3D gels. **A** and **B** Dose‒response viability testing of A549 cells with CTG after 72 h of treatment with iBET151. Cells were grown in either 2D (**A**) or 3D Matrigel (**B**). The IC50 values were calculated using the dose‒response nonlinear regression model from GraphPad Prism. The percentage of viability for p60AmotL2-expressing cells is represented by the blue line and is compared to control cells. From top to bottom, 95% CI for **A**: [10.39, 16.00] and [5.270, 6.647]; **B**: [38.92, 63.63] and [4.214, 5.917]; **C**: [15.74, 23.01] and [12.22, 15.20]; **D**: [35.59, 86.75] and [7.060, 16.28]. Data represent the mean ± SEM from three independent experiments

Next, we assessed the efficacy of validated compounds in A549 cells (lung adenocarcinoma) constitutively expressing p60AmotL2 by means of lentiviral infection, both in 2D and 3D culture conditions. Interestingly, p60AmotL2-expressing cells cultured in 2D conditions (Fig. 7A and C) showed a moderate decrease in cell viability when treated with BET inhibitors, with a 2.2-fold difference in relative IC50 values between p60AmotL2-positive cells and control cells for iBET151 and a 1.4-fold difference for iBET762; whereas in 3D cultures, there was a more accentuated decrease in cell viability upon treatment with BET inhibitors, with a 9.8-fold and a 4.9-fold difference in relative IC50 values for iBE151 and iBET762, respectively (Fig. 7B and D).

**Fig. 8:**
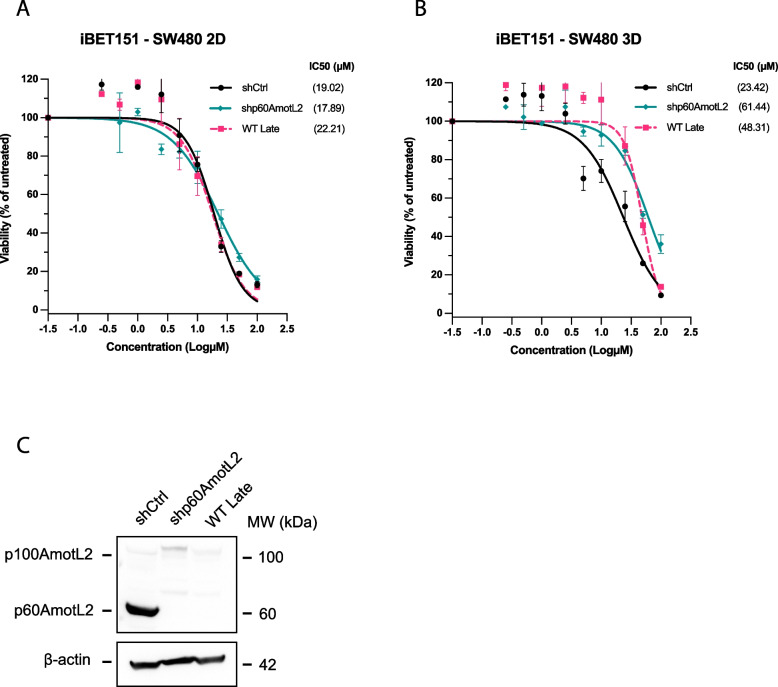
Sensitivity to BET inhibitors mediated by endogenous p60AmotL2. **A** and **B** Early passage shCtrl (p60AmotL2 $+$), shRNA p60AmotL2-depleted (p60AmotL2 $-$) or WT Late passage (p60AmotL2 $-$) SW480 cells treated with iBET151 in 2D (**A**) or 3D cultures **B**). The IC50 values were calculated using the dose‒response nonlinear regression model from GraphPad Prism. From top to bottom, 95% CI for **A**: [13.82, 26.38], [17.59, 28.08] and [13.51, 23.81]; for **B**: [17.33, 31.51], [51.81, 74.58] and [37.27, 63.53]. **C** Western blot analysis of SW480 colon cancer cells at early (shCtrl), p60AmotL2-depleted (shp60AmotL2) and late passage (WT Late) in cell culture. Data represent the mean ± SEM from three independent experiments

Then, we sought to evaluate the effectiveness of BET inhibitors in tumor cells that express endogenous p60AmotL2. To accomplish this, we employed SW480 cells, originally derived from a colorectal adenocarcinoma tumor. Notably, these cells exhibit the presence of endogenous p60AmotL2 at low passage numbers, a characteristic that quickly becomes attenuated as cells are cultured in vitro over successive passages. Additionally, we have previously described a targeted approach to selectively modulate the expression of p60AmotL2 in these cells [14]. The expression levels of p100 and p60AmotL2 in SW480 cells were examined using Western Blot analysis. It was observed that in late passages of SW480 cells, p60AmotL2 expression was no longer detectable (as shown in Fig. 8C). Similarly, the application of a shRNA specifically targeting the p60AmotL2 isoform resulted in a near-complete depletion of p60AmotL2.

In a similar fashion to MDCK and A549 cells, iBET151 showed an increased ability to inhibit control SW480 cells (shCtrl) with endogenous p60AmotL2 expression, when cultured in 3D conditions, compared to shp60AmotL2 or late passage (WT Late) SW480 cells lacking p60AmotL2 (Fig. 8A and B). In a 2D setting there was no apparent difference (Fig. 8A). Relative IC50 values between p60AmotL2-expressing (shCtrl) and p60AmotL2-depleted (shp60AmotL2) SW480 cells showed a 2.6-fold difference for 3D cultures. These results suggest that the use of 3D cultures may be a more accurate model for testing the efficacy of candidate drugs in vitro, as they better mimic the complex microenvironment found in vivo.

Subsequently, we aimed to evaluate the efficacy of BET inhibitors on patient-derived cells expressing p60AmotL2 in a 3D culture environment. For that purpose, patient-derived organoid (PDO) models represent a promising approach for screening cancer drugs [39, 40]. PDOs are three-dimensional structures derived from patient tumor tissues that can be grown in vitro or ex vivo. These structures resemble the architecture and microenvironment of the original tumor, making them an important model system for studying cancer and testing candidate drugs [41].

We then used PDOs derived from colon cancer patients who underwent surgery at Södersjukhuset, Stockholm, Sweden. Organoids were generated according to the protocol described in the Materials and Methods section. We evaluated the effects of iBET151 and iBET762 on colon cancer organoids derived from three patients. As previously mentioned, p60AmotL2 is not expressed under normoxic conditions [16], necessitating the introduction of exogenous p60AmotL2 using a lentiviral vector. The expression of p60AmotL2 was subsequently analysed by western blot, as detailed in Supplemental Data 5.

**Fig. 9:**
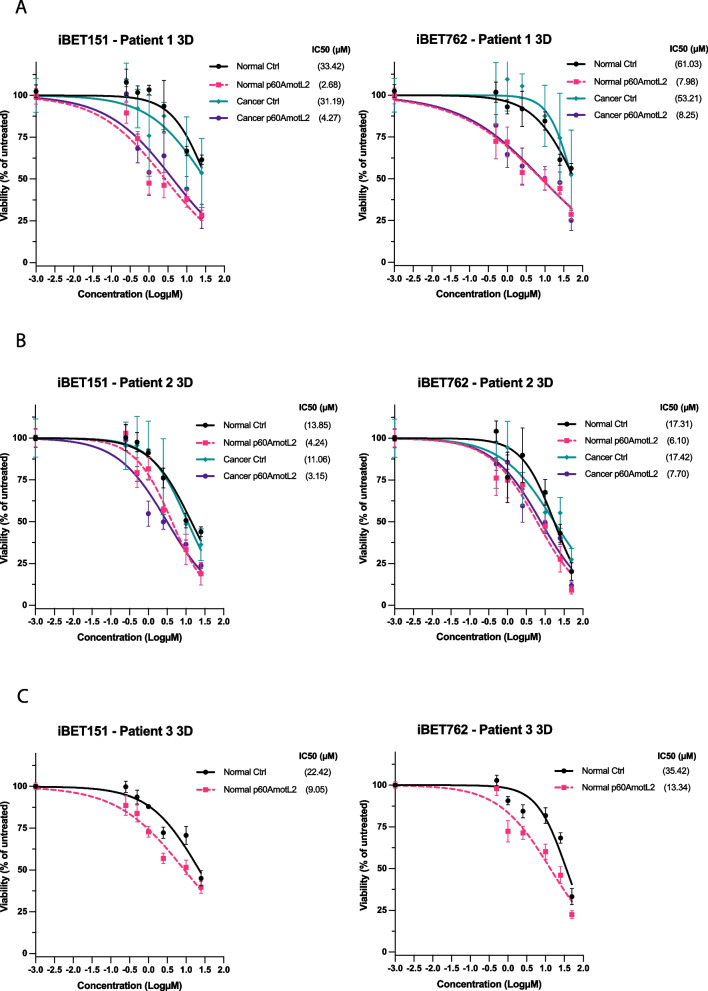
Efficacy of iBET151 and iBET762 on 3D Matrigel PDOs expressing p60AmotL2. **A**, **B**, and **C** Dose‒response viability testing with CTG after 72 h of treatment with iBET151 and iBET762 on PDOs derived from Patient 1 (**A**), Patient 2 (**B**), and Patient 3 (**C**), respectively. The IC50 values were calculated using the dose‒response nonlinear regression model from GraphPad Prism. The percentage of viability for p60AmotL2-expressing PDOs is represented by the pink and purple lines and is compared to control PDOs (black and green lines). From top to bottom, 95% CI for Patient 1 (**A**): iBET151 [22.26, 67.70], [1.709, 4.370], [17.18, 93.10], [2.085, 10.35] and iBET762 [41.89, 117.7], [4.408, 15.04], [39.59, 93.17], [4.425, 16.12]; Patient 2 (**B**): iBET151 [10.02, 20.52], [2.937, 6.264], [7.819, 16.54], [2.203, 4.644] and iBET762 [10.50, 28.52], [3.877, 9.456], [12.02, 26.67], [5.264, 11.30]; Patient 3 (**C**): iBET151 [15.92, 35.67], [6.208, 14.38] and iBET762 [28.83, 46.62], [9.561, 19.03]. Data represent the mean ± SEM from three independent experiments

The results presented in Fig. 9 demonstrate that PDOs derived from both paired Normal or Cancer tissue exhibit increased sensitivity to iBET151 and iBET762 upon expressing p60AmotL2. Specifically, p60AmotL2-expressing PDOs from Patient 1 displayed a 12.5-fold increase in sensitivity to iBET151 in Normal tissue-derived organoids and a 7.3-fold increase in Cancer-derived organoids, compared to non-expressing controls, as shown in Fig. 9A (left panel). Similarly, for iBET762, these PDOs exhibited a 7.6-fold increase when derived from Normal tissue and a 6.4-fold increase when derived from Cancer in terms of relative IC50 values, detailed in Fig. 9A (right panel).

PDOs from Patients 2 and 3 showed a more moderate response, correlating to a lower p60AmotL2 expression when compared with PDOs from Patient 1 (Supplemental Data 5). PDOs from Patient 2 treated with iBET151 had a 3.3-fold (Normal) and a 3.5-fold (Cancer) difference in relative IC50 values when comparing p60AmotL2-expressing cells to control cells (Fig. 9B, left panel); for iBET762 we had a 2.8-fold (Normal) and a 2.3-fold (Cancer) difference in relative IC50 values (Fig. 9B, right panel). Similarly, PDOs derived from Patient 3 showed a 2.5-fold (Normal) and a 2.7-fold (Normal) difference in relative IC50 values when treated with iBET151 (Fig. 9C, left panel) and iBET762 (Fig. 9C, right panel), respectively.

The sensitivity to BET inhibitors (BETi) appeared to correlate more closely with the level of p60AmotL2 expression rather than whether the PDOs were derived from either paired Normal or Cancer tissues. This observation is further supported by Fig. 6, which demonstrates that BETi-induced caspase-3 activation was primarily associated with p60AmotL2 expression levels. While Hepatocyte Growth Factor (HGF) is known to promote cell motility in cells such as MDCK [42], control cells treated with HGF, regardless of Doxycycline (Dox) presence, exhibited minimal cleaved caspase-3 staining in contrast to p60AmotL2-inducible cells treated with Dox alone or in combination with HGF.

### Nascent RNA sequencing after BETi

**Fig. 10:**
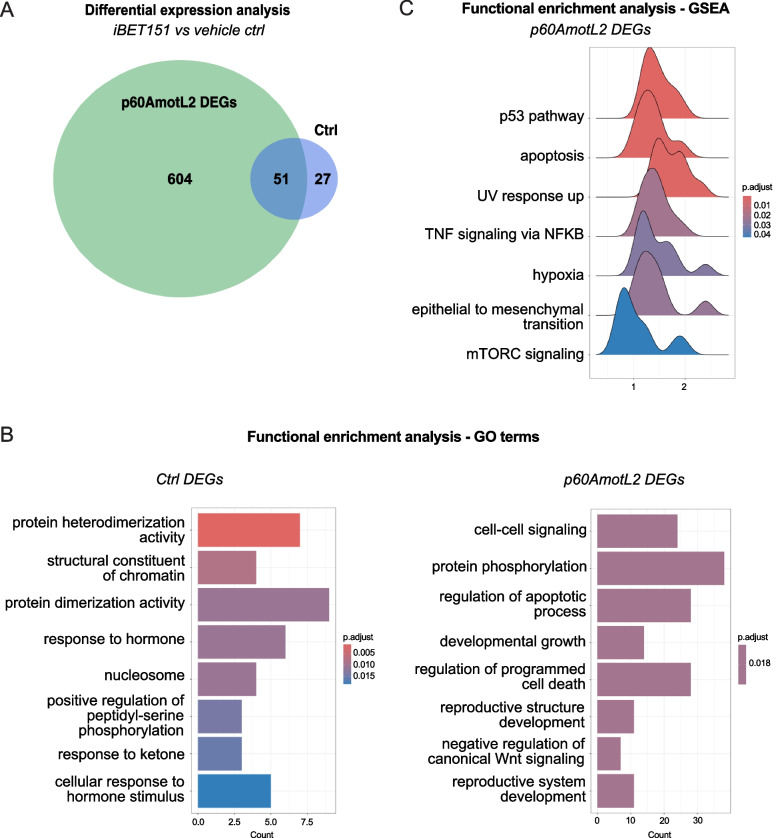
Nascent RNA sequencing revealed a p60AMOTL2-dependent transcriptional response to BET inhibition. **A** The Venn diagram illustrates the dependence of BET inhibitor-induced transcriptional change on p60AMOTL2, highlighting 655 differentially expressed genes (DEGs) in p60AmotL2-expressing cells in contrast to only 78 DEGs in control cells after iBET151 treatment, based on exon feature unique counts. An adjusted *p*-value cutoff < 0.1 was used. **B** and **C** Functional enrichment analyses of the DEGs in p60AmotL2-expressing and non-expressing cells after iBET151 inhibition for Gene Ontology (GO) terms and Gene Set Enrichment Analysis (GSEA), with significant hallmark gene sets found only in p60AMOTL2 expressing cells (< 0.05 p-adj, BH)), respectively. These analyses revealed a significant shift towards pro-apoptotic pathways associated with p53 signalling. An adjusted *p*-value cutoff < 0,05 was used

To elucidate the mechanisms behind the increased sensitivity of cells expressing p60AmotL2 to BET inhibitors, we performed nascent RNA sequencing following treatment with iBET151 using Global Run-On sequencing (GRO-seq). The dependence of iBET151-induced transcriptional response on the presence of p60AMOTL2 is illustrated on the Venn diagram of Fig. 10A, where cells expressing p60AmotL2 display 655 differentially-expressed genes (DEGs) following iBET151 treatment (indicated by the green circle), in stark contrast to the mere 78 DEGs observed in control cells (depicted in blue).

Subsequent functional enrichment analyses of the DEGs were conducted to analyse the immediate transcriptional effects of BET inhibition in the presence or absence of p60AmotL2. Gene Ontology (GO) and Gene Set Enrichment Analysis (GSEA), as depicted in Fig. 10B and C, respectively, revealed a significant shift towards pro-apoptotic pathways associated with p53 signaling specifically in p60AMOTL2-expressing cells. This observation correlated with the increased cell death observed in p60AmotL2-expressing cells after BET inhibition.

## Discussion

Here we present a novel strategy to target cell invasion using phenotypic screening of low molecular weight compounds. The rationale for our approach is based on the evidence supporting the pivotal role of p60AmotL2 in tumor progression. This evidence is based on patient expression data, where we have shown that p60AmotL2 is predominantly expressed in invasive fronts of various epithelial cancers. Moreover, functional validation from prior studies utilizing cellular and animal models has established that p60AmotL2 expression correlates with an increased invasive potential [13, 14].

However, a significant hurdle in the mechanistic understanding of p60AmotL2 action is the downregulation of p60AmotL2 expression under normoxic conditions in vitro [16], which complicated the use of functional assays. This issue was particularly evident in the SW480 colon carcinoma cell line, which exhibited robust p60AmotL2 expression in early passages but showed a marked loss of expression with successive culturing. Consequently, we designed a doxycycline-inducible system to reintroduce p60AmotL2 into our cell lines and organoid models, with a particular focus on MDCK cells as the primary target for initial screening. The reasoning for selecting MDCK cells for the initial screen is based on their well-documented characteristics, stable phenotype and ability to polarize and form 3D structures in vitro. Interestingly, our investigations have unveiled that p60AmotL2 induces a shift in epithelial cells from collective to amoeboid migration and invasion [14]; a mode of cell movement frequently observed in cancer cells and often associated with aggressive and invasive tumor phenotypes [43].

While two-dimensional cell cultures have long been the basis of most drug screening assays, they come with inherent limitations regarding screening for cell invasion inhibitors. Despite these constraints, we opted for a 2D approach for the initial screening with subsequent hit validation in 3D cultures, as analysis of the proteome showed that p60Amotl2 induced protein expression consistent with an invasive phenotype even in 2D.

Our screening results revealed that cells expressing p60AmotL2 displayed heightened sensitivity to EGFR and BET inhibitors. Notably, while p60AmotL2 did not appear to affect EGF-receptor activation levels, our previous research has suggested that it disrupts apical polarity and the cell sorting of membrane receptors such as c-Met [13]. This disruption in cellular compartmentalization may provide an explanation for the observed sensitivity. It is important to note, however, that this heightened sensitivity to EGF receptor tyrosine kinase inhibitors was not replicated in other human cancer cells we tested. EGFRi could also not replicate the inhibitory effects on p60AmotL2-expressing cells when cells were treated after reaching confluency or a plateau phase and, as a result, were not further investigated in this study. It is conceivable that in this scenario, cellular adaptations modulate the response to receptor tyrosine kinase inhibitors (RTKi), necessitating the use of combination therapy to overcome resistance [44]. For instance, the strategy of using RTKi in combination with BETi in the treatment of ovarian cancer has been investigated as a way to overcome RTK signaling adaptations [45].

Another compelling discovery from our study relates to BETi. These inhibitors represent a distinctive class of epigenetic modifiers known to selectively bind the bromodomains of Bromodomain and Extra-Terminal motif (BET) proteins, namely BRD2, BRD3, BRD4, and BRDT. These inhibitors effectively disrupt protein‒protein interactions between BET proteins and acetylated histones, as well as transcription factors, thereby affecting gene transcription in oncogenic networks, which are often dependent on BET proteins [46]. Notably, several investigations have also underscored the critical roles of BET proteins in orchestrating large-scale chromatin structure rearrangements [47].

BET inhibitors are currently used to treat patients with NUT midline carcinoma, driven by a BRD4-NUT fusion oncoprotein [48], and are a potential treatment avenue for tumors that have aberrant BET protein or gene function, such as ameloblastoma [49]. Additionally, even in cancers with no apparent BET protein or gene aberrations, the antagonistic effect of BETi in the binding of BET proteins to acetylated histones can serve to epigenetically suppress anti-apoptotic and cancer-promoting genes. Moreover, BETi treatment often leads to the upregulation of pro-apoptotic genes, either indirectly or via inhibiting BRD4-mediated repression through the NuRD complex [50–52]. Currently, BET inhibitors are undergoing investigation in clinical trials for a wide spectrum of cancer types [53–55].

Our research has underscored the observation that BET inhibitors exhibit a preference for targeting invading p60AmotL2-expressing cells, irrespective of whether they are normal or transformed cells. Our previous data have shown that the main isoform of AmotL2, p100AmotL2, is exclusively expressed in normal cells, while the shorter isoform, p60AmotL2, has only been observed in cancer cells; the exception being that it is also transiently expressed in non-malignant and redundant cells being extruded by the process of apical extrusion [16]. This may help explain why BET inhibitors seem to selectively target tumor cells over normal cells in a clinical setting [46], as the former show more aggressive and invasive phenotypes than the latter.

Nonetheless, there are some reservations with using BETi to treat cancer due to dose-limiting toxicities when used as a single agent [56]. This is hardly surprising, as BETi can affect proteins with a general role in transcriptional initiation and/or elongation, potentially affecting regular or homeostatic gene transcription. However, ongoing clinical trials point to the use of these inhibitors in combination therapies aiming to prevent and revert drug resistance [57, 58]. Moreover, BETi are also being investigated for their potential in blocking oncogenic super-enhancers [59], with the idea that these inhibitors will work in blocking whatever cells are aberrantly overexpressing. This highlights the potential of using BETi to treat not only cancer, but also other diseases that arise due to aberrant expression of certain genes or proteins [60, 61].

BETi have the ability to target a multitude of cancer-related pathways, such as MYC [62], P53 [51] and YAP signaling [63]. In this study, the exact molecular mechanisms by which BETi selectively target p60AmotL2-expressing invasive cancer cells remain to be elucidated. We have previously demonstrated that p100AmotL2 transmits force from cellular junctions to the nuclear lamina, playing a crucial role in maintaining nuclear localization and integrity [14, 15]. More recently, we have shown that p60AmotL2 functions as a dominant-negative variant of its larger isoform, p100AmotL2, leading to the disconnection of the nuclear lamina from the cytoskeleton [14]. Considering that p60AmotL2-expressing cells show altered nuclear mechanical properties, as shown by previous atomic force microscopy experiments [14], we argue that these invasive cells have distinguished chromatin and epigenetic landscapes which might explain the heightened sensitivity to BETi.

Supporting this hypothesis, a recent study from our lab has shown that impairing the AmotL2 signaling axis by using short hairpin RNAs—which is phenocopied by p60AmotL2 via acting as a dominant-negative of the main isoform—leads to reduced chromatin accessibility [20]. While the mechanism of decreased chromatin accessibility in p60AMOTL2-expressing cells is not explored here, a role for perturbed cytoplasmic contractile actin filaments [17] cannot be ruled out. Cytoplasmic actin dynamics has thus indirect effects on nuclear processes by influencing the availability of actin monomers [64]—a rate limiting step in actin nuclear import. In turn, nuclear actin has well-established roles in regulating chromatin remodeling and the activity of specific transcription factors, supports transcription by RNA polymerases I, II and III and facilitates RNA processing [64].

To investigate the transcriptional basis for the differential sensitivity to BET inhibition, we employed nascent RNA sequencing to delineate active transcriptional profiles. Bioinformatics analyses revealed a stronger dependence of transcriptional processes on BET protein functions in the presence of p60AmotL2 genome wide. Subsequent differential expression analysis indicated that most of the DEGs are predominantly involved in pathways related to apoptosis and p53 signalling.

We thus propose that BET inhibition of p60AmotL2-expressing invasive cancer cells leads to a shift towards pro-apoptotic p53-related signaling pathways, ultimately leading to increased cell death relative to iBET151-treated control cells.

Further studies are needed to elucidate the exact mechanisms of action and the time-dependent association between chromatin accessibility, global transcriptional dependencies on BET proteins, actin dynamics and cell death in p60AmotL2-expressing invasive cancer cells after treatment with BETi.

## Conclusion

In conclusion, our study presents a promising and innovative approach to combat cell invasion by using low molecular weight compounds identified via phenotypic screening. Our findings demonstrate that BET inhibitors are particularly effective against invasive cells expressing p60AmotL2. We propose that the expression of p60AmotL2 enhances invasive capabilities in concert with epigenetic landscape alterations, rendering tumor cells more susceptible to BET inhibition. This insight underscores the potential of employing BET inhibitors as combination therapies against invasive cancers in a clinical setting, offering novel therapeutic options for patients with advanced metastatic diseases. This underlines the need for further research on understanding the molecular mechanisms by which BET inhibitors work in targeting cancer cells. We hypothesize that p60AmotL2-expressing invading cancer cells have altered chromatin dynamics and chromatin accessibility relative to normal cells, which in turn makes these cells more susceptible to BETi-induced cell death, potentially by p53-related pro-apoptotic signaling pathways.

These findings also highlight the potential for future drug screening approaches, especially with using larger compound libraries to discover innovative agents capable of exploiting different chromatin or epigenetic dynamics and effectively combatting invasive cancers.

### Supplementary Information

**Supplementary Material 1. **

**Supplementary Material 2. **

**Supplementary Material 3. **

**Supplementary Material 4. **

**Supplementary Material 5. **

**Supplementary Material 6. **

## Data Availability

The datasets (RNA-seq, Proteomics, Drug Screening) supporting the conclusions of this article will be made available in a repository.
