# Supplementary material for: A phenotypic screening approach to target p60AmotL2-expressing invasive cancer cells
Source: J Exp Clin Cancer Res. 2024 Apr 9;43:107. doi: 10.1186/s13046-024-03031-w (PMC11003180; doi:10.1186/s13046-024-03031-w)
Supplement: Supplementary file 4 — Supplementary Material 4. [file 13046_2024_3031_MOESM4_ESM.docx]

**Supplemental Data 4. Table with sDSS values for 60 candidate compounds from primary and validation phenotypic drug screens.** Compounds are sorted based on sDSS values from primary screen, highest to lowest, and were divided into two tables, RTKi and BETi (and others). Compounds with a validated sDSS above 4.5 are highlighted in bold.

**Supplemental Data 5. Western blots show p60AmotL2 expression in MDCK p60AmotL2-inducible cells.** Western blots for A549 p60AmotL2-constitutive cells, SW480 p60AmotL2-endogenous cells, and PDOs constitutively expressing p60AmotL2.

**Supplemental Data 6. Patient materials and details.**

| **Patient** | **Age** | **Gender** | **Tumor type** | **TNM stage** | **Specifications & comments** |
| --- | --- | --- | --- | --- | --- |
| No.1 | 80 | female | High grade adenocarcinoma | pT4aN1b | Tumor in right side flexure. |
| No.2 | 86 | male | High grade adenocarcinoma | pT4bN1a | Tumor in transverse colon. Subtotal colectomy. MSI medullary colon cancer. |
| No.3 | 84 | female | High grade adenocarcinoma | pT4aN1a | Right sided hemicolectomy. |
