# Supplementary material for: A phenotypic screening approach to target p60AmotL2-expressing invasive cancer cells
Source: J Exp Clin Cancer Res. 2024 Apr 9;43:107. doi: 10.1186/s13046-024-03031-w (PMC11003180; doi:10.1186/s13046-024-03031-w)

Supplemental Data 8

Full blots for Supplemental Data 5

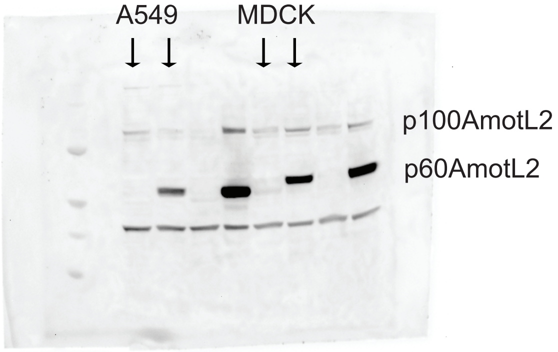

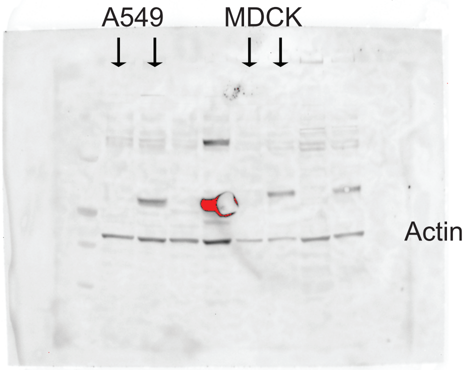

Patient 1

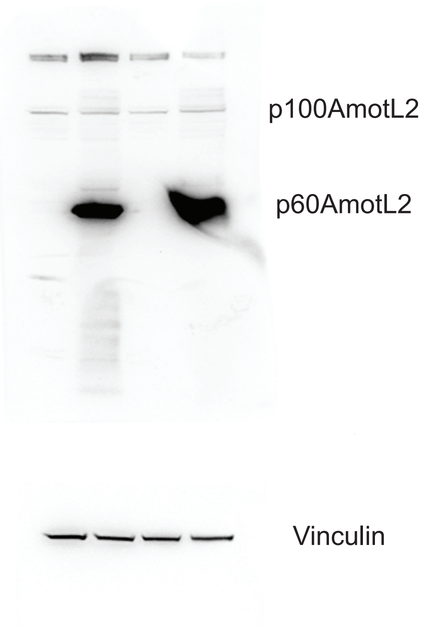

Patient 2

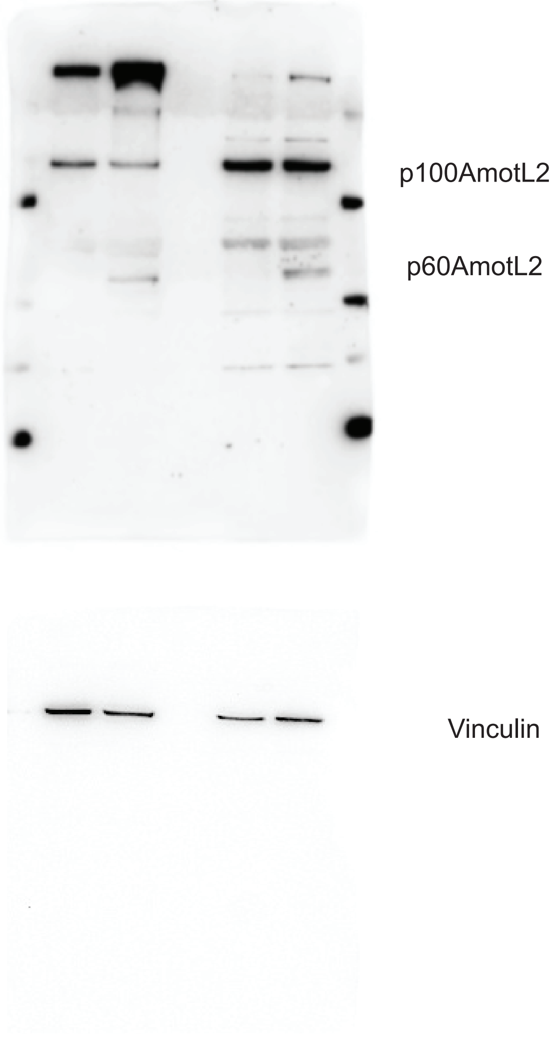

Patient 3

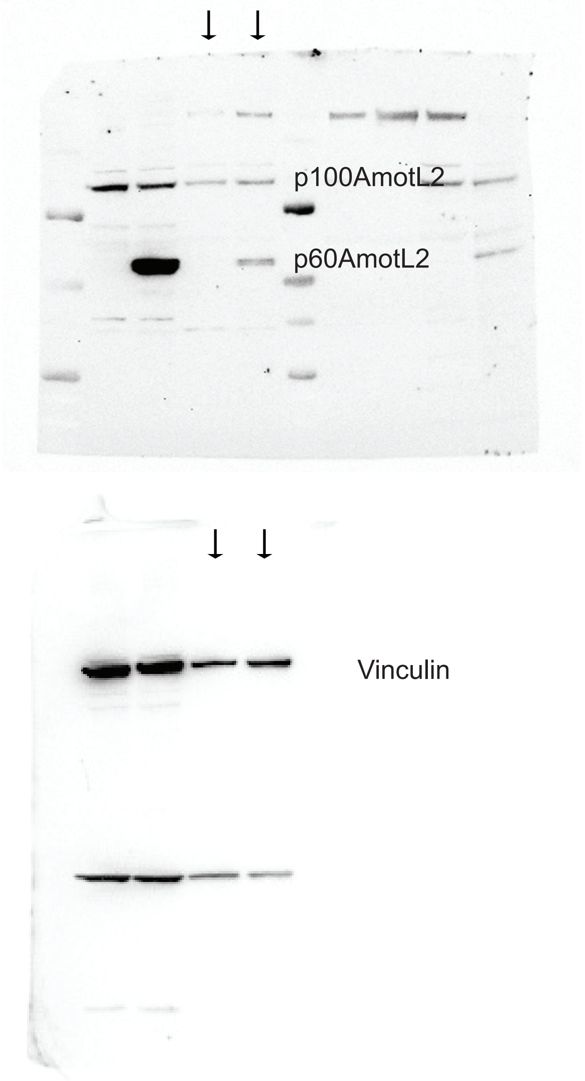

Full blots for Figure 8

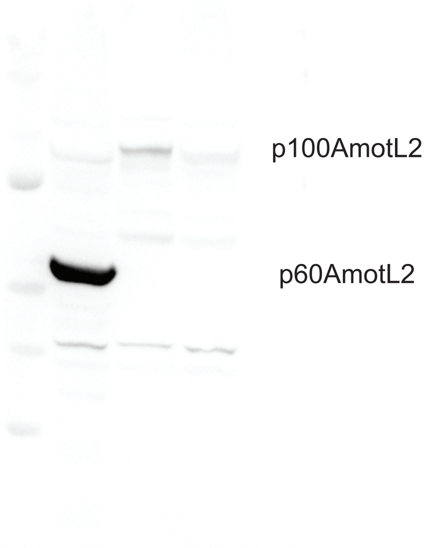

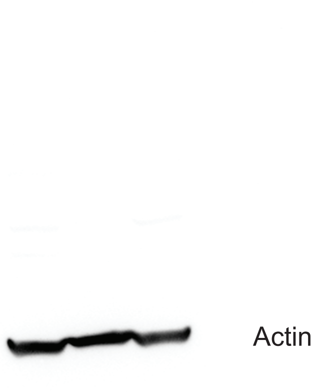

Supplement: Supplementary file 6 — Supplementary Material 6. [file 13046_2024_3031_MOESM6_ESM.pdf]
